# Automated chick gender determination using optical coherence tomography and deep learning

**DOI:** 10.1016/j.psj.2025.105033

**Published:** 2025-03-15

**Authors:** Jadsada Saetiew, Papawit Nongkhunsan, Jiraporn Saenjae, Rapeephat Yodsungnoen, Amonrat Molee, Sirichok Jungthawan, Ittipon Fongkaew, Panomsak Meemon

**Affiliations:** a School of Physics, Institute of Science, Suranaree University of Technology, 111 University Avenue, Muang, Nakhon ratchasima 30000, Thailand; b School of Integrated Science and Innovation, Institute of Science, Suranaree University of Technology, 111 University Avenue, Muang, Nakhon ratchasima 30000, Thailand; c School of Animal Technology and Innovation, Institute of Agricultural, Suranaree University of Technology, 111 University Avenue, Muang, Nakhon ratchasima 30000, Thailand

**Keywords:** Chick gender classification, Vent sexing, Optical coherence tomography, Deep learning, Machine learning

## Abstract

Chick gender classification is crucial for optimizing poultry production, yet traditional methods such as vent sexing and ultrasound remain limited by human expertise, labor intensity, and insufficient resolution. This study introduces a novel approach that integrates Optical Coherence Tomography (OCT) and deep learning to enable high-resolution, non-invasive chick sexing. Unlike conventional imaging techniques, OCT provides micrometer-scale visualization of cloacal structures, allowing precise differentiation between male and female chicks based on internal anatomical markers. We developed a custom convolutional neural network (CNN) optimized for OCT data, incorporating asymmetric image resizing and enhanced feature extraction to improve classification accuracy. Our model achieved 79 % accuracy, outperforming conventional architectures such as Inception (63 %) and VGG-16 (74 %), highlighting the importance of a tailored, domain-specific model. This is the first study to integrate OCT with deep learning for automated chick sexing, demonstrating a scalable, real-time alternative to expert-dependent vent sexing. With further advancements in imaging and machine learning, our approach has the potential to transform chick sexing in commercial hatcheries, reducing reliance on skilled labor while enhancing classification efficiency and precision.

## Introduction

In recent years, breeding chickens with specific traits, such as gender, has become increasingly important for poultry farmers worldwide. In many regions, farms prioritize female chicks due to their value in egg production, while in some farming systems, both male and female chickens are raised, e.g. males primarily for meat, and females for both meat and eggs. For example, in the production of Korat chickens in Thailand, farmers tend to favor male chickens for their faster growth rates and higher-quality meat. In cases where both sexes are raised together, farms may face reduced meat quality if gender selection is not optimized (Kaleta & Redmann, 2008). Consequently, raising chickens of a specific gender can lead to significant cost savings. However, accurately determining the gender of newly hatched chicks poses a challenge, as both sexes exhibit similar characteristics that only become distinctly identifiable after approximately one month.

Traditional gender classification methods, such as feather and cloaca examination, are widely used. The color of down feathers is a common indicator, but it becomes unreliable as chickens mature. Another widely known method, "vent sexing," proposed by Masui and Hashimoto (1993), involves inspecting the chick's cloaca to determine its gender (Masui & Hashimoto, 1933). This method relies on identifying small anatomical differences: males have a cone-shaped structure below the intestinal opening, while females have a smaller, hemisphere-shaped structure. However, both feather and cloaca-based methods have an accuracy of around 80%, primarily due to human error, dependency on expert skill, and inter-operator variability. Since vent sexing requires years of training and is labor-intensive, scaling this method for large poultry farms remains challenging.

In efforts to reduce human error, researchers have explored transcutaneous ultrasonography to examine the internal anatomy of chickens. However, detecting small organs like testes and ovaries remains difficult due to variations in tissue reflection, limiting the effectiveness of this method for gender determination. While ultrasonography has been successfully used to assess ovarian status in hens and predict the number of large yellow follicles, its reliability in identifying smaller reproductive structures is significantly lower (Melnychuk et al., 2002). Additionally, although ultrasonography can detect gross ovarian tumors, its sensitivity decreases for smaller or early-stage abnormalities, further restricting its application (Barua et al., 2007).

X-ray and MRI imaging offer high-resolution visualization of internal structures but are impractical for large-scale commercial hatcheries due to cost, processing time, and operational constraints. X-ray imaging requires ionizing radiation, raising safety concerns and necessitating stringent regulatory compliance. MRI, while providing detailed soft tissue contrast, is expensive, time-consuming, and demands specialized equipment and personnel, making its widespread adoption in hatcheries unrealistic (Streckenbach et al., 2024).

Hyperspectral imaging (HSI) has been explored for biological tissue differentiation but is limited by its shallow penetration depth, making it unsuitable for visualizing deeper anatomical structures like the cloaca, which is essential for accurate chick gender determination. Studies show that near-infrared HSI light can penetrate only a few millimeters into biological tissues, restricting its ability to capture subsurface anatomical features necessary for sex classification (Huang et al., 2016). Furthermore, HSI's effectiveness in detecting structural differences has been primarily validated in surface-level applications, which do not extend to deeper tissue assessment (Kho et al., 2019).

Deep learning-based methods using vocalization or visible light imaging have shown promise but face limitations due to the minimal external morphological differences between male and female chicks. While vocalization-based classification techniques leverage audio features to differentiate sexes, their accuracy depends on subtle sex-specific vocal characteristics, making them inconsistent (Li et al., 2022). Similarly, facial image-based classification struggles due to the lack of distinguishable visual markers between sexes in the visible spectrum (Veganzones Rodriguez et al., 2024). Recent deep learning studies analyzing vocalization waveforms, zero-crossing rates, and signal durations have reported accuracy rates ranging from 74.55 % to 76.15 %, with overall performance averaging above 87 % (Cuan et al., 2022). Although these results demonstrate the potential of AI-driven solutions, they remain constrained by reliance on external, rather than internal, anatomical features.

Despite advancements in imaging and machine learning, existing chick sexing methods still pose significant challenges. Vent sexing, while widely used, remains dependent on highly trained personnel, making large-scale implementation difficult. Ultrasound imaging lacks the resolution required to distinguish small cloacal structures, while X-ray and MRI imaging, though highly detailed, are cost-prohibitive and impractical for high-throughput hatcheries. Similarly, hyperspectral imaging, while beneficial for surface differentiation, does not provide the necessary depth penetration for cloacal structure analysis. These limitations underscore the need for a high-resolution, real-time, and non-invasive imaging technique capable of improving classification accuracy while reducing reliance on expert labor.

Optical coherence tomography (OCT) emerges as a promising solution for chick gender classification, offering high-resolution cross-sectional imaging through non-invasive, near-infrared light (Huang et al., 1991). There are two main types of OCT: Time-domain OCT (TD-OCT) and Spectral-domain OCT (SD-OCT) (Fercher et al., 1995). TD-OCT relies on temporal interference of a broadband light source, but its speed is constrained by the need for mechanical scanning of a reference mirror to generate depth-sectioning images (Bouma & Tearney, 2002). In contrast, SD-OCT operates in the spectrum domain, eliminating the need for mechanical scanning and allowing for much faster imaging (Cense et al., 2004; Fercher et al., 2003).

Compared to other imaging techniques, OCT was chosen for its unique advantages in chick gender classification. Unlike ultrasound, OCT provides micrometer-scale resolution, allowing for precise identification of anatomical differences between male and female cloacas. Unlike MRI and X-ray imaging, OCT is radiation-free, cost-effective, and suitable for real-time analysis, making it a viable option for large-scale poultry farms. Furthermore, its non-contact nature reduces stress on chicks during classification. By capturing internal structural variations that are not visible with standard imaging methods, OCT presents an ideal solution for chick sexing applications.

OCT has been widely applied across various fields, including dermatology (Lee et al., 2011; Rolland et al., 2013), ophthalmology (Schmoll et al., 2009; Wojtkowski et al., 2005; Zvietcovich et al., 2019), biology (Davis et al., 2008; Tearney et al., 1996), aquatic toxicology (Bellini et al., 2014; Nanthanawat et al., 2024; Thanomsit et al., 2022, 2025), and material characterization (Meemon et al., 2013; Yao et al., 2015). Recent studies highlight its potential for classification tasks beyond biomedical applications. For example, research on gender classification using spatio-frequency feature fusion of OCT fingerprint images in the IoT environment demonstrates that OCT imaging can capture intricate structural details suitable for machine learning-driven classification (Kong et al., 2024). Additionally, OCT has been integrated into robotic systems for high-precision imaging and manipulation, as seen in robotic-OCT guided inspection and microsurgery of monolithic storage devices (He et al., 2023). These developments align with the objectives of this study, which seeks to integrate OCT with deep learning to automate chick gender classification, reducing reliance on human expertise while enhancing scalability for poultry farming.

This study explores the potential of OCT for real-time chick gender classification. An in-house Spectral Domain OCT (SD-OCT) system was developed to capture high-resolution images of cloacal tissue structures, providing detailed anatomical insights for classification. Through laboratory and field experiments, we demonstrated that these images offer sufficient detail for accurate gender differentiation. To minimize human error and reliance on skilled personnel, we integrated OCT with computer vision and deep learning techniques, developing a customized convolutional neural network (CNN)-based classification model in Python. Our approach achieves 79 % accuracy, comparable to traditional vent sexing, but with added advantages of automation, reduced processing time, and scalability for high-throughput hatcheries. Unlike previous studies that rely on vocalization-based classification or external morphological features, this study leverages high-resolution internal imaging of OCT, making gender classification more precise and anatomically informed. Additionally, we explored preprocessing optimizations, such as asymmetric image resizing and data augmentation, which further improved classification accuracy, offering a new perspective on OCT-based deep learning applications. With further advancements in OCT imaging technology and deep learning algorithms, this approach has the potential to offer a transformative, scalable, and non-invasive solution for the global poultry industry.

## Materials and methods

### Animal preparation

This study used newly hatched chicks of the Thai indigenous crossbred chicken, known as the 'Korat chicken,' to evaluate the efficacy of Optical Coherence Tomography (OCT) imaging for gender identification. A total of 30 chicks (15 males and 15 females) were selected from a local hatchery at Suranaree University of Technology. The chicks were handled following ethical guidelines for animal research, ensuring minimal stress and appropriate care during the imaging procedures. The protocol was reviewed and approved by the Ethics Committee on Animal Use at Suranaree University of Technology, Nakhon Ratchasima, Thailand, under document ID U1-02631-2559.

### Preparation of subjects for OCT imaging

Before imaging, each chick was gently restrained in a custom-made holding apparatus designed to ensure stability and comfort. Minimal feather displacement was performed manually to provide unobstructed access to the cloaca, which was then cleaned with a sterile saline solution to remove debris or contaminants. No sedation or invasive procedures were required due to the non-contact nature of the OCT system. This standardized protocol ensured reliable data collection across all samples, forming a robust foundation for subsequent analysis.

### OCT system for data collection

**Fig. 1:**
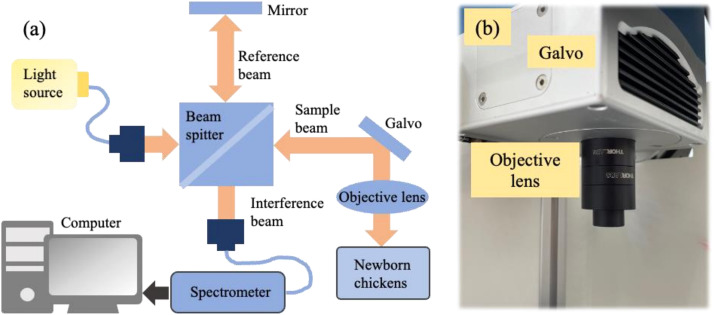
(a) Schematic diagram of the optical setup used for spectroscopic imaging of newborn chickens. The setup includes a light source, beam splitter, objective lens, and spectrometer for data collection. (b) A photograph of the physical system showing the galvo scanner and the objective lens used for focusing light onto the target.

The OCT imaging system was designed for portability, cost-efficiency, and compactness, making it suitable for field applications. As shown in Fig. 1, the spectral-domain OCT (SD-OCT) system employed a super luminescent light-emitting diode (SLED) (BSLD-850-10SM-FA, Box Optronics Technology Co., Ltd., China) as the light source, with a central wavelength of 840 nm, achieving a depth resolution of approximately 10 microns.

A free-space Michelson interferometer split the light into reference and sample beams via a beam splitter cube (CCM1-BS014/M, Thorlabs Inc., USA). The reference beam was directed to a retro-reflective mirror (PS975-B, Thorlabs Inc., USA), while the sample beam was focused on the cloaca using an objective lens (AC254-035-B-ML, Thorlabs Inc., USA). Reflected light was collected by the same lens, and the interference signal was recombined at the beam splitter.

The resulting interference signal was transmitted through a second single-mode fiber optic to the detection unit, where a high-speed spectrometer processed it. A Fourier transform was applied to generate depth scan signals. A galvanometer mirror (SG72208, Sino-Galvo Technology Co., Ltd., China) enabled beam scanning to produce cross-sectional images at a resolution of 1000 × 1000 pixels, covering a 4 mm x 4 mm area with a lateral sampling distance of 4 µm per pixel. The system operated at 25 frames per second.

For each OCT cross-sectional image, a scale factor adjustment procedure was applied to normalize the OCT signal intensity. As a standard procedure in all SD-OCT systems, each depth-resolved cross-sectional image was constructed from the envelope of the Fourier-transformed signal. The maximum amplitude of the detected envelope was identified as the reference scaling factor, which was then used to normalize the signal by dividing all intensity values by this factor. This process ensured a uniform intensity distribution across all images, correcting for variations in acquisition conditions. When necessary, additional contrast normalization was applied to enhance anatomical feature visibility. Finally, a logarithmic transformation was performed to compress the wide dynamic range of the data, allowing it to fit into an 8-bit grayscale image format.

### Vent sexing using OCT imaging

To ensure comprehensive anatomical representation for chick gender classification, 50 high-resolution OCT images were captured per chick from different locations around the cloacal region. This multi-location imaging and data augmentation approach were designed to account for anatomical variations and enhance the model's ability to generalize across different samples. From the 50 captured images per chick, 40 images were manually selected for deep learning training. The selection process was based on image clarity, structural visibility, and anatomical relevance, ensuring that only high-quality images were used for classification. As a result, the final dataset used for model training and evaluation consisted of 1200 high-resolution OCT images (30 chicks × 40 images each).

**Fig. 2:**
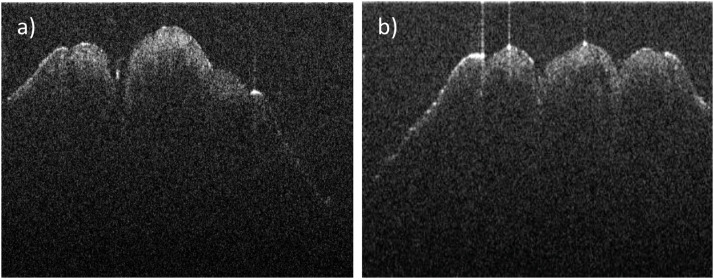
OCT cross-sectional images of newly hatched chicks cloaca: (a) female and (b) male.

**Fig. 3:**
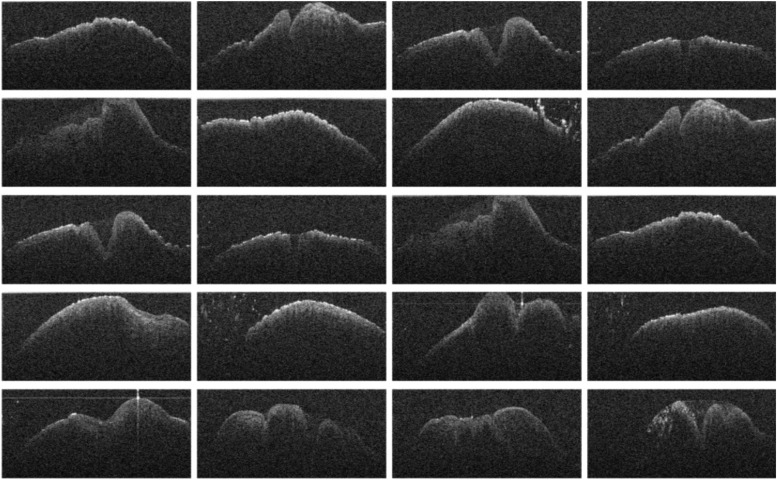
Series of OCT cross-sectional images of newly hatched female chicks' cloaca without venting.

**Fig. 4:**
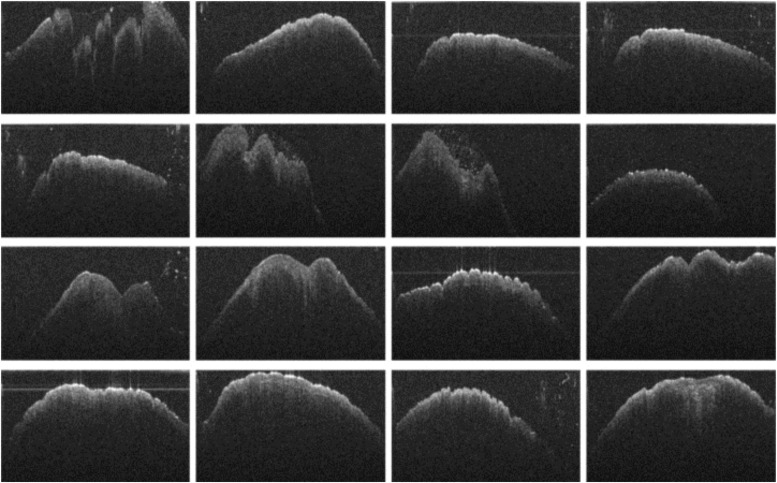
Series of OCT cross-sectional images of newly hatched male chicks' cloaca without venting.

Grayscale OCT images (1000 × 600 pixels) clearly distinguished male and female chicks, as shown in Fig. 2. Male chicks displayed a phallus with three cones (Fig. 2b), while female chicks showed only two (Fig. 2a). This differentiation enabled straightforward gender identification with near-perfect accuracy in preliminary tests. To improve efficiency, cloacal images were also captured without physical venting to reduce handling stress on the chicks. As shown in Fig. 3, Fig. 4, Raw OCT imaging data often contained noise and artifacts, which could negatively impact deep learning model performance. To mitigate this, preprocessing techniques, including noise reduction, contrast enhancement, and image sharpening, were applied to enhance image quality.

### Deep learning

**Fig. 5:**
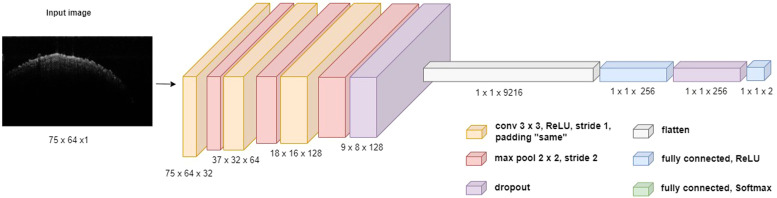
A schematic representation of the CNN architecture used for sex classification of newborn chickens based on OCT image input. The network consists of multiple convolutional layers with ReLU activations, followed by max-pooling and dropout layers. The extracted features are passed through fully connected layers for classification into male or female categories.

A convolutional neural network (CNN) was implemented to automate and enhance chick gender classification using OCT images. To compensate for the relatively small number of individual chicks, additional data augmentation techniques were applied, including rotation and flipping to simulate imaging variations and improve feature recognition, contrast and brightness normalization to account for differences in tissue reflectivity, and asymmetric image resizing to optimize spatial feature extraction for deep learning analysis. All OCT images were converted to grayscale and resized to 75 × 64 pixels to balance computational efficiency while preserving critical anatomical details. The dataset was split into 80% for training (960 images) and 20% for testing (240 images), ensuring a reliable evaluation framework. As shown in Fig. 5, the custom convolutional neural network (CNN) developed for chick gender classification consists of the following layer-by-layer architecture:

• **Input Layer:** Accepts grayscale OCT images resized to 75 × 64 pixels (1 channel).
• **Conv1:** 32 filters, 3 × 3 kernel, stride 1, padding ‘same’, ReLU activation (Agarap, 2018).
• **MaxPool1:** 2 × 2 pool size, stride 2, reducing spatial dimensions to 37 × 32.
• **Conv2:** 64 filters, 3 × 3 kernel, stride 1, padding ‘same’, ReLU activation.
• **MaxPool2:** 2 × 2 pool size, stride 2, reducing spatial dimensions to 18 × 16.
• **Conv3:** 128 filters, 3 × 3 kernel, stride 1, padding ‘same’, ReLU activation.
• **MaxPool3:** 2 × 2 pool size, stride 2, reducing spatial dimensions to 9 × 8.
• **Dropout:** 25% dropout rate to prevent overfitting (Srivastava et al., 2014).
• **Flatten:** Converts 3D feature maps into a 1D vector (9 × 8 × 128 = 9216 features).
• **Fully Connected (FC1):** 256 units, ReLU activation.
• **Dropout:** 50 % dropout rate (Srivastava et al., 2014).
• **Output Layer:** 2 units (male/female), softmax activation for binary classification.

The model was trained using the Adam optimizer with a learning rate of 0.0001, a batch size of 128, and 1000 epochs, with early stopping applied if validation loss did not improve for 50 epochs.

### Evaluation

The performance of the model was evaluated using a confusion matrix, which provided key metrics such as precision, recall, accuracy, and the F1-score. The trained model was tested against the test dataset, and the confusion matrix helped to quantify its performance. Additionally, we compared our model's efficiency with other pre-trained models, such as InceptionV3 (Szegedy et al., 2016) and VGG16 (Simonyan & Zisserman, 2014), both trained on the ImageNet-1K dataset. A summary of the performance evaluation is presented in Table 1.

**Table 1:** Confusion matrix results for the validation dataset.

	Female	Male	Not detect
Female	352	58	0
Male	109	274	2
Not detect	1	0	27

## Results

**Fig. 6:**
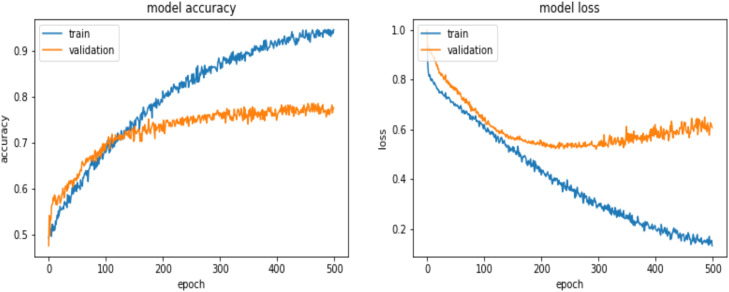
Training and validation performance of the convolutional neural network model. (Left) The model accuracy over 500 epochs, showing an increase in training accuracy with a slower rise in validation accuracy. (Right) The model loss over 500 epochs, indicating a decrease in training loss and a stabilization of validation loss, suggesting convergence and potential overfitting in later epochs.

This study utilized image classification based on OCT scans to determine the gender of newly hatched chicks. Using the data obtained from the deep learning training process, a preliminary evaluation was conducted. The learning curve visually represents the model's performance, with the x-axis indicating the number of epochs and the y-axis showing metrics such as loss and accuracy. As shown in Fig. 6, the model performed optimally with fewer than 150 epochs, suggesting that deep learning can make accurate predictions based on the testing data. At 150 epochs, both the accuracy and loss for the training and validation datasets were well-fitted, indicating good model performance.

The confusion matrix, shown in Table 1, summarizes the model's classification results. This matrix provides valuable insights into how well the model predicted chick gender, allowing for the calculation of key statistical metrics such as precision, recall, and F1-score providing a statistically robust evaluation of the model's classification performance and reflecting consistent prediction reliability across the test dataset, which are presented in Table 2. The proposed model (Model 1) achieved an accuracy of 79 %, outperforming the conventional InceptionV3 model (63 %) and the VGG-16 model (74%). Our model, based on a modified VGG architecture, demonstrated that reducing the number of convolutional layers and incorporating a dropout layer can enhance accuracy for classifying OCT images. The results highlight that our model outperformed both the Inception and VGG-16 models across key metrics. The modifications made to the VGG-based architecture, including the reduction of convolution layers and the introduction of dropout, significantly improved performance. While Model2 (Inception) and Model2 (VGG-16) are sophisticated architectures, their performance here reveals specific insufficiencies. Inception's multi-scale inception modules, designed for diverse feature extraction, may dilute focus on the localized anatomical differences (e.g., three cones in males vs. two in females) critical for OCT-based gender classification. Similarly, VGG-16’s deep, uniform structure (16 layers) excels in hierarchical feature extraction for natural images but is less efficient and adaptable for our grayscale, high-resolution OCT images, contributing to its lower accuracy. Model 1’s tailored design prioritizes these task-specific features, explaining its superior performance. This comparison highlights Model 1’s efficacy against established models, though it is limited to these two benchmarks to provide a clear baseline.

**Table 2:** The performance of proposed model based on various image size.

Image size (pixels)	Precision		Recall		F1-score		Accuracy
	macro	weighted	macro	weighted	macro	weighted	
1000 × 600 (baseline)	0.8394	0.7973	0.8448	0.7934	0.8397	0.7920	0.7934
224 × 224	0.6757	0.6120	0.6355	0.6102	0.6538	0.6108	0.6102
128 × 128	0.7369	0.6553	0.6950	0.6539	0.7142	0.6544	0.6539
128 × 64	0.7700	0.6724	0.7559	0.6667	0.7614	0.6676	0.6667
64 × 64	0.6954	0.6764	0.7422	0.6776	0.7156	0.6758	0.6776
75 × 64	0.7203	0.6987	0.7920	0.6995	0.7511	0.6985	0.6995

Additionally, we examined the impact of image size on the model's performance during training and inference. Larger image sizes require more computational resources, which can be costly and time-consuming. Table 2 presents the performance of the model using various image sizes, showing that smaller image sizes led to unexpected improvements. For instance, the model's performance dropped by approximately 18% when the image size was reduced from the baseline 1000 × 600 pixels to 224 × 224 pixels. However, performance increased again with further reductions in image size, reaching 67 % accuracy at 64 pixels. Interestingly, an asymmetric image size of 75 × 64 pixels outperformed the 64 × 64 pixel size by about 2 %, indicating that using asymmetric images may be more effective for chick gender classification.

## Discussion

The high-resolution images provided by OCT were crucial for distinguishing subtle differences between male and female cloacas, such as the number of cones in the phallus. This non-invasive method offers a reliable alternative to vent sexing, reducing the risk of error and the need for skilled personnel. The ability to automate this process has significant implications for scalability and efficiency in poultry farming.

Table 3 illustrates that our OCT-based approach achieves accuracy comparable to traditional vent sexing (79 % vs. ∼80 %) while offering non-invasiveness and high scalability, advantages shared with vocalization-based methods but with the added benefit of direct anatomical imaging.

**Table 3:** Comparison of the proposed OCT-based method with existing chick gender classification techniques based on accuracy, invasiveness, scalability, and reliance on expertise.

Method	Accuracy (%)	Invasiveness	Scalability	Expertise Required	Reference
Vent Sexing	∼80	Invasive	Low	High	(Masui & Hashimoto, 1933)
Ultrasonography	Variable	Non-invasive	Moderate	Moderate	(Barua et al., 2007; Melnychuk et al., 2002)
Vocalization (CNN)	74.55–87	Non-invasive	High	Low	(Cuan et al., 2022; Li et al., 2022)
Hyperspectral Imaging	Not specified	Non-invasive	Moderate	Moderate	(Huang et al., 2016; Kho et al., 2019)
OCT + CNN (This Study)	79	Non-invasive	High	Low	-

**Fig. 7:**
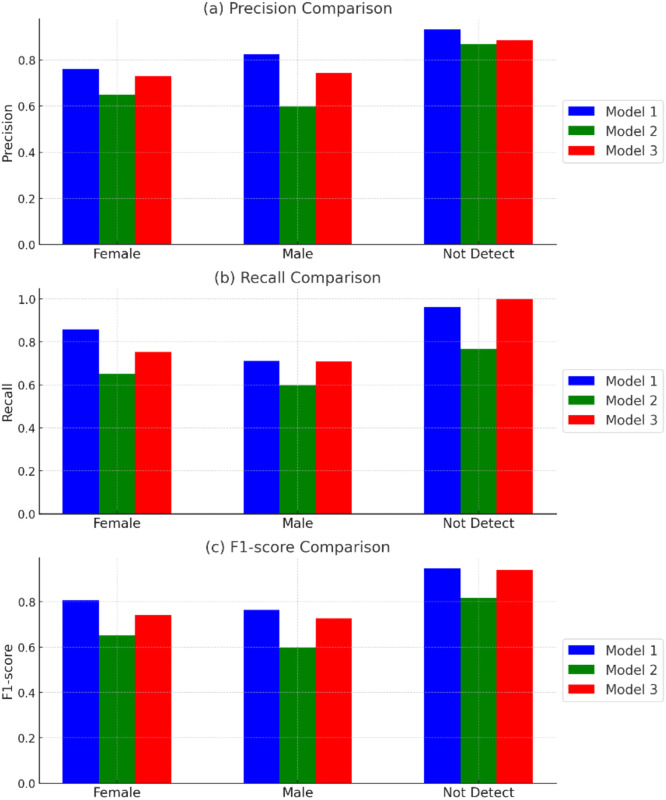
Performance comparison of three models (Model 1, Model 2, and Model 3) across different metrics for sex classification. (a) Precision comparison for "Female," "Male," and "Not Detect" categories, showing variations in the models' ability to correctly identify true positives. (b) Recall comparison, highlighting the models' sensitivity in detecting true positives across the categories. (c) F1-score comparison, demonstrating the harmonic mean of precision and recall for each model and category.

Our proposed model (Model1) achieved an accuracy of 79 %, outperforming Model2 (Inception) at 63 % and Model3 (VGG-16) at 74 %, underscoring the limitations of conventional architectures for chick gender classification using OCT images. While Inception is highly effective for complex RGB datasets due to its multi-scale feature extraction via inception modules, it proves less suitable for this task. Its broad feature extraction approach may overlook subtle, localized cloacal structures, such as the three cones in males versus two in females (Fig. 2), which are captured in our grayscale OCT scans. Fig. 7

Similarly, VGG-16, despite its deep architecture and extensive convolutional layers, is constrained by its computational complexity and pretraining on diverse natural images from ImageNet. This general-purpose design struggles to adapt to the high-resolution, domain-specific OCT data, resulting in reduced accuracy and efficiency compared to Model1.

In contrast, our custom CNN, featuring a streamlined architecture with fewer convolutional layers, ReLU activations, dropout for regularization, and asymmetric image resizing (75 × 64 pixels), is optimized for detecting these critical anatomical markers. This highlights that for specialized tasks like OCT-based chick gender classification, a targeted, simpler model can outperform more complex, generalized architectures. While our comparison is limited to these benchmarks, the results emphasize the importance of designing models tailored to domain-specific imaging data.

Further enhancing Model1’s performance, we found that image size significantly influences classification outcomes, impacting precision, recall, and F1-score (Table 2). Larger images (e.g., 1000 × 600 pixels) offer rich detail, improving feature extraction and accuracy but at the expense of higher computational demands and longer processing times. Conversely, smaller symmetric sizes (e.g., 64 × 64 pixels) reduce resource needs and accelerate inference, yet they sacrifice fine-grained details, potentially compromising performance. Notably, an asymmetric size of 75 × 64 pixels outperformed the 64 × 64-pixel configuration by approximately 2 %, indicating that preserving specific spatial dimensions enhances the model's ability to discern gender-specific features. This finding underscores the importance of tailoring preprocessing steps, such as image resizing, to balance computational efficiency with classification accuracy. These insights highlight how strategic optimization can refine deep learning models for practical applications, such as real-time gender determination in poultry farming, offering a scalable alternative to traditional methods.

This study demonstrates that combining OCT with deep learning is a promising alternative to traditional vent sexing for determining the gender of newborn chicks. Our model achieved an accuracy of 79 %, comparable to manual methods but with the added advantages of reducing human error and reliance on specialized skills.

While our proposed model achieves 79% classification accuracy, which is comparable to traditional vent sexing methods, this level of precision may still be inadequate for large-scale industrial deployment, where minimizing errors in chick sorting is critical. Even small misclassification rates can lead to significant economic losses for poultry farms, particularly when precise sex selection is essential for optimizing production. Enhancing model accuracy remains a key objective for future research.

Although this study demonstrates feasibility using a controlled dataset of 30 Korat chicks, external validation with an independent cohort from diverse breeds and hatcheries was not conducted due to laboratory constraints. This limitation affects the immediate generalizability of the model. However, the use of data augmentation and a balanced train-test split supports its robustness within the tested population. Future studies should prioritize external validation to confirm model performance across varied conditions.

Expanding the dataset to include more chick samples from various breeds and environmental conditions is one of the most effective strategies for improving accuracy. A larger dataset would expose the model to a broader range of anatomical variations, leading to better generalization and more reliable classification across different populations. Additionally, implementing semi-supervised or active learning approaches could refine predictions using high-confidence examples, reducing reliance on manual labeling while enhancing model adaptability.

Enhancing image quality and feature extraction is another crucial factor. Increasing the resolution of OCT scans could help capture finer anatomical details that may currently be overlooked, improving the deep learning model's ability to differentiate between male and female cloacas. Furthermore, incorporating advanced image preprocessing techniques, such as adaptive histogram equalization, denoising filters, and edge enhancement, could further optimize image quality, enhancing feature recognition and classification accuracy.

With further refinement, this approach has the potential to streamline gender classification in commercial hatcheries, offering real-time, accurate, and scalable solutions. Future research should focus on expanding the dataset with a larger number of chick samples across different breeds and environmental conditions to improve model generalization. Additionally, incorporating advanced deep learning architectures, such as ResNet, DenseNet, and transformer-based models, could enhance feature extraction and classification performance. Efforts should also be made to optimize real-time implementation, ensuring that model predictions remain both accurate and computationally efficient for practical use in high-throughput hatchery environments. Furthermore, exploring multi-modal classification techniques that integrate various imaging and non-imaging data sources, such as hyperspectral imaging, thermal imaging, and vocalization analysis, could provide complementary features that improve classification robustness. By implementing these strategies, we anticipate that the accuracy of OCT-based deep learning models for chick gender classification can be significantly improved, making them viable for large-scale industrial deployment.

## Conclusion

In conclusion, this study demonstrates that the combination of Optical Coherence Tomography (OCT) and deep learning techniques is a promising approach for automating chick gender classification. By utilizing high-resolution OCT images, we were able to capture detailed structural differences between male and female chicks, which significantly improved the accuracy of gender determination. Our custom deep learning model achieved an accuracy of 79 %, which is comparable to traditional vent sexing methods but with the added benefits of reduced human error and the potential for large-scale automation. This suggests that integrating OCT with machine learning can provide a scalable and efficient solution for poultry farming operations.

Furthermore, our exploration of the impact of image size on model performance revealed that careful optimization is key to maintaining a balance between accuracy and computational efficiency. While larger images provided higher precision and recall, smaller asymmetric images (e.g., 75 × 64 pixels) surprisingly yielded competitive results, indicating that certain spatial dimensions may be more crucial than overall image size. These findings highlight the importance of tailoring preprocessing steps, such as image resizing, to the specific needs of the task at hand. Future research should focus on further refining the OCT imaging process and deep learning algorithms to enhance performance and explore how these techniques can be applied to other areas of animal classification and diagnostics.

## Disclosures

The authors declare the following financial interests/personal relationships which may be considered as potential competing interests:

Panomsak Meemon reports financial support was provided by 10.13039/501100017170Thailand Science Research and Innovation. Jadsada Saetiew reports financial support was provided by National Science, Research, and Innovation Fund. If there are other authors, they declare that they have no known competing financial interests or personal relationships that could have appeared to influence the work reported in this paper.
